# Morphometrics of the coronoid process and the radial notch of the ulna: implications for fracture assessment

**DOI:** 10.1007/s00276-023-03249-x

**Published:** 2023-10-14

**Authors:** Krishnan Sircar, Nikolaus Kernich, Martin Scaal, Peer Eysel, Lars-Peter Müller, Tim Leschinger

**Affiliations:** 1 Department of Orthopedics and Trauma Surgery, Faculty of Medicine and University Hospital Cologne, University of Cologne, Kerpener-Strasse 62, 50937 Cologne, Germany; 2 Faculty of Medicine, Institute of Anatomy II, University of Cologne, Joseph-Stelzmann Str. 9, 50937 Cologne, Germany

**Keywords:** Coronoid process, Anatomy, Radial notch, Sigmoid notch, Proximal radioulnar joint, Fracture

## Abstract

**Purpose:**

A comprehensive analysis of the morphology of fractures of the coronoid process (CP) can aid diagnosis and guide treatment. The involvement of the radial notch of the ulna (RN)—e.g., in anterolateral facet fractures and transverse fractures of the CP—may influence the biomechanical conditions of the proximal radioulnar joint. However, the morphometric relation between the CP and the RN and the extent to what the proximal radioulnar joint can be affected in these types of fractures is unknown.

**Methods:**

A total of 113 embalmed, cadaveric ulnae were dissected. All soft tissue was removed. Strictly lateral, high-resolution photographs were taken and digitally analyzed. The height of the CP and its relation to the RN was measured. Sex differences and correlations between measured parameters were calculated.

**Results:**

Mean height of the CP was 16 mm (range: 12–23 mm; SD: 2). Mean height of the RN was 16 mm (11–25 mm; 2.2). The 50% mark of the CP corresponded to 18% (0–56%; 11.2) of the height of the RN. No significant differences were found between male and female specimens.

**Conclusion:**

The RN of the ulna extends only to a small part to the CP. Transverse or anterolateral fractures of less than 50% of the coronoid process may involve only a small portion of the proximal radioulnar joint.

## Introduction

**Fig. 1:**
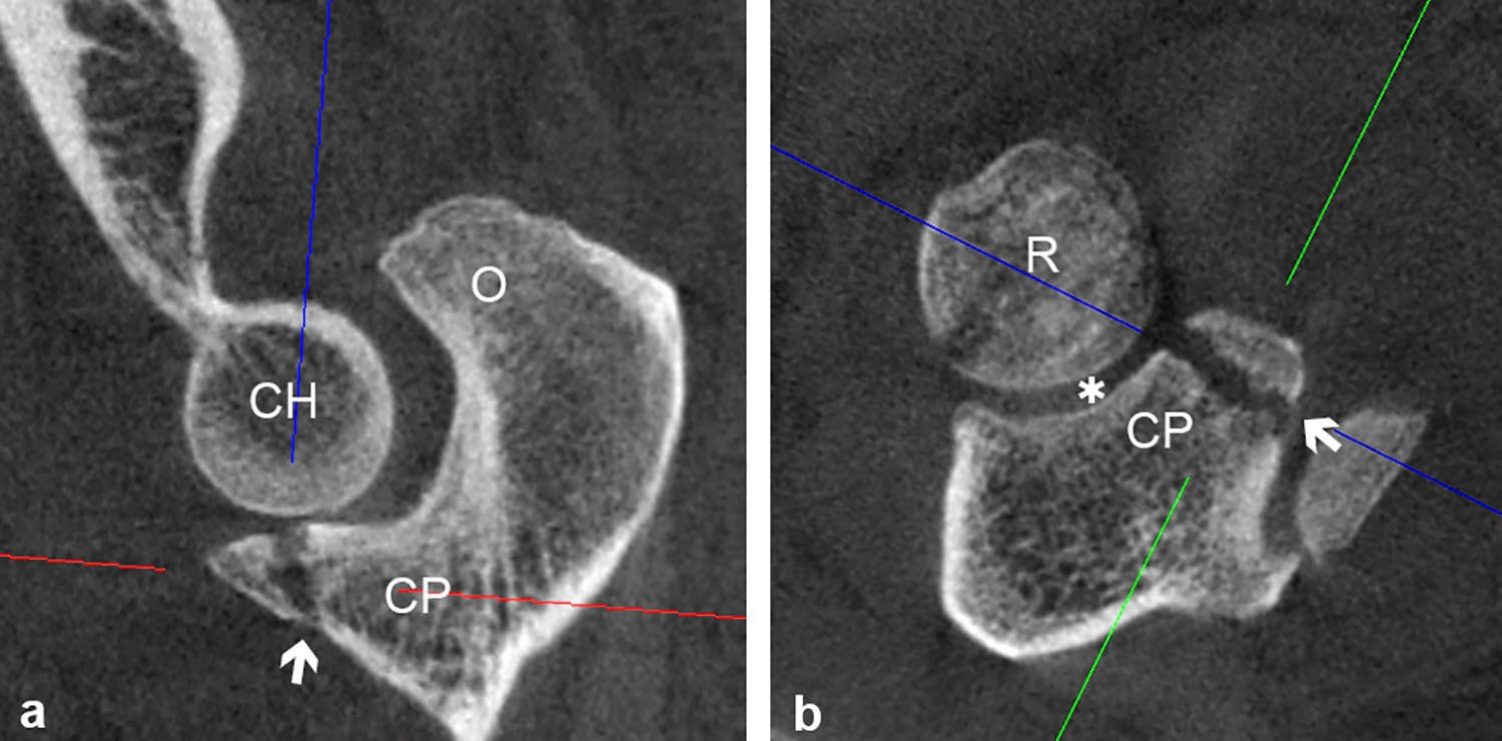
**a**, ** b** CT scan (**a**: sagittal view; **b**: axial view) of an elbow joint of a 52-year-old male with an anterolateral fracture of the coronoid process and fracture of the radial head. The white arrows denote the fracture line. The fracture line corresponds to approximately 50% of the height of the coronoid process (Regan and Morrey Type 2 fracture) and also involves the proximal radioulnar joint (white star). *CP* coronoid process, *R* radial head, *CH* capitulum humeri, *O* olecranon

The coronoid process (CP) of the ulna is an important stabilizer of the elbow joint. Biomechanical and clinical studies have highlighted the crucial role of the coronoid process as an anterior buttress in the elbow joint, preventing joint dislocation. In 1989, Reagan and Morrey [16] introduced a classification system for coronoid fractures, primarily based on the assessment of fracture sizes through lateral radiographs. This initial classification was an important step in understanding and categorizing these fractures. However, in 2003, O’Driscoll et al. [13] recognized the need to consider not only fracture size but also the anatomical location and injury pattern of coronoid fractures. Their work emphasized the importance of a comprehensive evaluation that considers these additional factors to guide appropriate treatment strategies. This classification system illustrated the mechanism of injury associated with the anteromedial facet. Subsequent studies have identified another type of fracture, known as anterolateral fracture. This specific type of fracture is observed in coronoid tip fractures in terrible triad injuries (TTIs). The fracture lines are located in a relatively lateral position. Adams et al. [2] described that the coronoid injury patterns should also include oblique fractures of the coronoid anterolateral facet, accounting for approximately 7% of cases. They called for further studies to validate the feasibility of recognizing this new type of fracture. Moreover Rhyou et al. [17] also found that patients with combined coronoid fractures and radial head injuries often exhibited involvement of the coronoid anterolateral facet. This type of anterolateral or oblique lateral fractures can include the radial notch (RN), which forms the ulnar articular surface of the proximal radioulnar joint (Fig. 1 a, b). So far, the extent to which the CP constitutes the RN and the articular surface of the proximal radioulnar joint has not been studied. Apart from instability, the assessment of articular involvement of fractures is essential for decision making for or against surgical treatment. Therefore, the aim of this anatomic study is to analyze the relationship of the RN to the height of the CP. This could help to assess the articular involvement of anterolateral, oblique lateral or transverse CP fractures, improve diagnostics and guide treatment decisions.

## Materials and methods

**Fig. 2:**
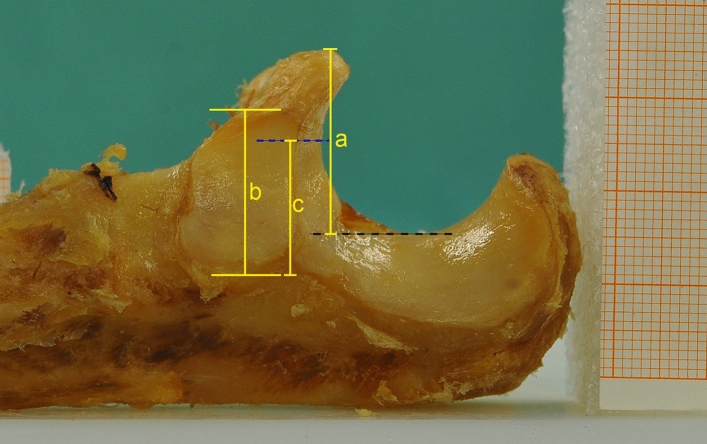
Exemplary lateral photograph of a specimen. Distance “a” measures the height of the coronoid process (CP) from the black dashed line (ridge of the trochlear notch) to the tip of the CP. Distance “b” measures the depth of the radial notch. The blue dashed line marks 50% of distance “a”. Distance “c” measures the distance from the posterior end of the radial notch to the 50% mark of the coronoid process

A total of 170 formalin-embalmed ulnae were screened for this study. The specimens were obtained through our university’s body donor program. Prior to testing, approval from our local ethics committee was obtained (ruling No. 16–040). Fifty-seven specimens were excluded due to visible damage of the cartilage and/or presence of osteophytes at the proximal ulnar. The remaining 113 ulnae were used for the study. The mean age of donors was 79 years (range: 61–102). Forty-three donors were male and seventy were female. All soft tissues were carefully dissected from the bone. Specimens were propped in an orthograde position next to a millimeter scale, and high-resolution pictures (300 dpi) were taken with a digital camera (Nikon D3, 60 mm focal length, f/10 aperture, 1/100 s exposure time, ISO 1000 film speed). To ensure parallax-free images, the camera was positioned in a strictly horizontal position on a tripod and on the same level as the specimens. Images were digitally analyzed with ImagePro Plus 6 (Media Cybernetics). The following measurements were taken (Fig. 2):

- The height of the CP, measured from the tip of the CP to the ridge of the trochlear notch.
- The anterior–posterior depth of the RN, measured from the posterior end to the anterior end of the cartilaginous, articular surface.
- The distance from the posterior end of the RN to the 50% mark of the height of the CP.

**Fig. 3:**
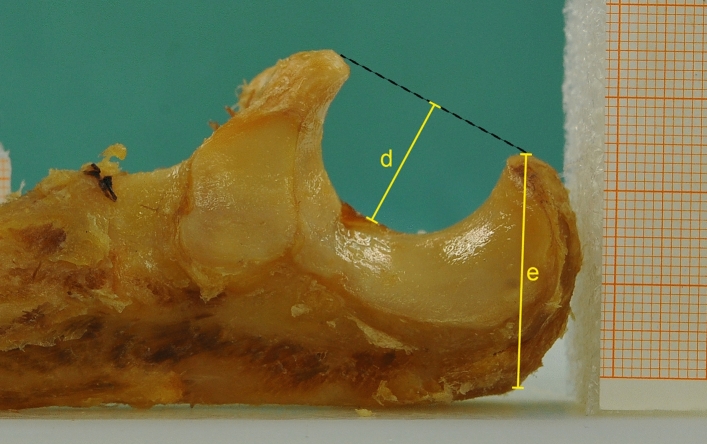
Exemplary lateral photograph of a specimen. Distance “d” measures the depth of the trochlear notch: a line (dashed black line) is drawn from the tip of the olecranon to the tip of the coronoid process and a second line (d) perpendicular to the first line is drawn to the deepest point of the trochlea. Distance “e” measures the height of the olecranon from its tip to its most posterior border

In addition, the depth of the trochlear notch (d) [9] and the height of the olecranon (e) [18] were measured as depicted in Fig. 3.

Measurements were independently taken by two investigators (KS and NK). Interrater agreement was calculated with Cohen’s Kappa and showed a high agreement (*ĸ* = 0.91). The measured data are presented as mean (range; ± standard deviation; [95% confidence interval]). The ratio of c to b was then calculated and is presented as percentage in the form of mean (range, ± standard deviation; [95% confidence interval]). Sex-specific differences were analyzed with Student’s *t* test and level of significance was set at *p* < 0.05. To assess correlations between the measured parameters, the Pearson coefficient (*ρ)* was calculated.

## Results

The mean height of the CP was 15.8 mm (12.2–23.3 mm; ± 2.0; [15.4–16.2]). The mean depth of the RN was 15.6 mm (10.6–25.0 mm; ± 2.2; [15.1–16]). The mean distance from the posterior end of the RN to the 50% mark of the CP was 12.7 mm (7.4–20.7 mm; ± 1.9; [12.4–13.1]). This corresponded to a mean of 18% (0–56%; ± 11; [16–20]) of the height of the RN.

The mean depth of the trochlear notch was 11.5 mm (10.2–13.7 mm; ± 1.3; [10.6–12.3]). The mean height of the olecranon was 24.5 mm (22.4–28.5 mm; ± 1.8; [23.3–25.7]).

Table 1 shows sex-specific results. There were no statistically significant differences between male and female specimens.

**Table 1:** Results of the measurements, stratified by sex and presented as mean in mm (range in mm, ± SD)

	Males (*n* = 40)	Females (*n* = 70)	*p* value
Height of CP^a^	16.2 (12.3–23.3; ± 2.4)	15.6 (12.2–19.3; ± 1.7)	0.2
Depth of RN^b^	15.6 (10.6–20.7; ± 2)	15.6 (10.8–25; ± 2.4)	0.9
Posterior end of RN to 50%-mark of CP	12.8 (8.9–20.7; ± 2.3)	12.7 (7.4–17.6; 1.7)	0.7
Height of olecranon	25.2 (22.8–28–5; ± 2.2)	24 (22.4–26.5; ± 1.4)	0.3
Depth of trochlear notch	11.7 (10.4–13–7; ± 1.6)	11.4 (10.2–13.6; ± 1.2)	0.8

^a^Coronoid process

^b^Radial notch

Table 2 shows the correlation between measured parameters.

**Table 2:** Correlation between measured parameters. Correlation is calculated as Pearson coefficient *ρ*: *ρ* ≥ 0.1 is considered a weak correlation, *ρ* ≥ 0.3 is a moderate correlation, and *ρ* ≥ 0.5 or higher is a strong correlation

Parameter pairing	*ρ*
Height of CP^a^–depth of RN^b^	0.5
Height of CP–depth of RN to 50% mark of CP	0.2
Height of CP–height of olecranon	0.7
Height of CP–depth of trochlear notch	0.8
Depth of RN–height of olecranon	0.5
Depth of RN–depth of trochlear notch	0.4

^a^Coronoid process

^b^Radial notch

## Discussion

In this anatomic study, we aimed to analyze the morphometric relationship between the coronoid process and the radial notch of the ulna in a large sample size. We found that the lateral aspect of the CP is only formed by a small fraction of the RN.

**Fig. 4:**
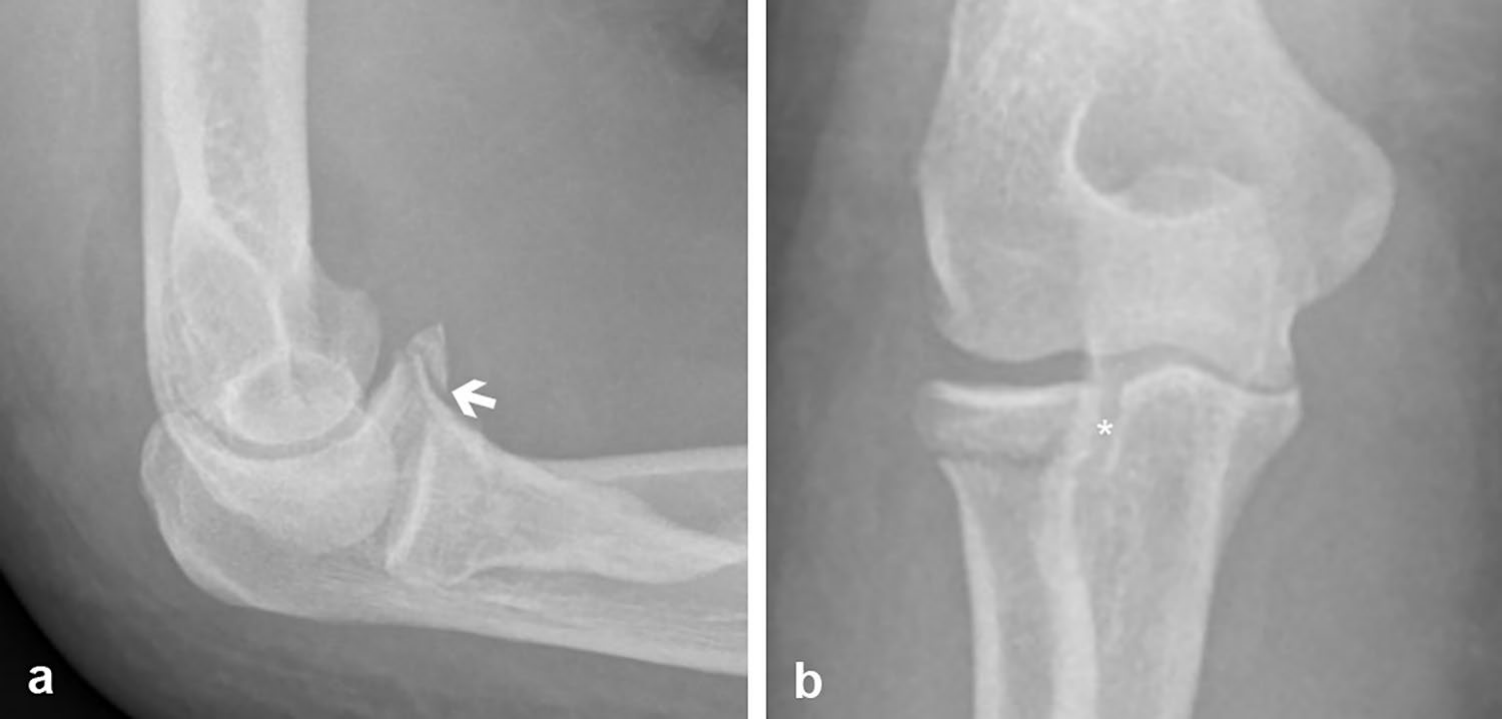
**a**,** b** Lateral (**a**) and anterior–posterior (**b**) radiographs from the same patient as in Fig. 1 a, b. Based on the radiographs, the fracture of the coronoid process (white arrow) may be classified as Regan and Morrey Type 1 or Type 2. The articular involvement of the radioulnar joint (white star) is not apparent from the radiographs alone, but requires a CT scan (Fig. 1 a, b)

Isolated fractures of the CP are rare. They are more commonly found in combination with other injuries of the elbow, such as dislocation, fractures of the radial head, and rupture of collateral ligaments. Although several classifications of CP fractures have been published, optimal treatment based on those classifications is still controversial [6, 19]. The first classification was developed by Regan and Morrey in 1989 [16]. They defined three types of fractures based on the fragment size in lateral radiographs. Type 1 corresponds to an avulsion of the tip of the CP, Type 2 is a fracture of up to 50% of the CP, and Type 3 is a fracture of more than 50% of the CP. It has been suggested that un-dislocated Type 1 and 2 fractures may be treated conservatively if there is no instability, radial head fracture or lateral ligament injury (or if the latter two are surgically repaired) and patients are followed-up closely [3, 4, 7, 14]. Fragment size according to the Regan and Morrey classification is also correlated to post-injury range of motion with larger fragments leading to a poorer range of motion [1]. While still used and simple to apply, other classifications have been developed to overcome the shortcomings of the Regan and Morrey classification. O’Driscoll et al. proposed a classification of three fracture types based on fracture patterns in 2003 [13]. It is closely related to injury mechanisms [5]. Type 1 is a fracture of the tip of the CP—similar to Regan and Morrey’s Type 1. Type 2 is a fracture of the anteromedial facet of the CP and Type 3 is a fracture through the base of the CP. While anteromedial fractures are generally considered to necessitate operative treatment, conservative treatment has been proposed for smaller fragments in the absence of dislocation or elbow subluxation [3]. Base fractures of the CP are usually treated surgically [10]. When assessing fractures, the amount of joint involvement is essential information. The articular surface of the RN, which forms the ulnar part of the proximal radioulnar joint, however, is not specifically considered in above classifications. Adams et al. [2] suggested a new classification based on 52 computer tomographies and first also included anterolateral fractures as a separate entity. Studies that focus on fractures involving the RN are rare. In a retrospective analysis of 72 patients with CP fractures, Rausch et al. [15] found a RN involvement in 29% of cases. However, the exact localization of the fracture within the RN and to what amount the RN was fractured was not examined. Mellema et al. [11] studied 110 computed tomographies of CP fractures and found an involvement of the proximal radioulnar joint in 92% of cases, predominantly at the anterior half of the RN. The extent of the involvement of the RN again remained unclear. Using quantitative three-dimensional computed tomographies of sixteen O’Driscoll Type 3 fractures, Kachooei et al. [8] showed that these types of fractures involve 42% of the surface of the RN. To the best of our knowledge, this is the first study that aims to analyze the morphometric relationship between the CP and the RN. We chose 50% of the CP height as our reference point as this corresponds to Regan and Morrey Type 2 fractures. This reference point corresponded to a mean of 18% of the height of the RN. This finding suggests that from an anatomic point of view, fractures that are classified as Regan and Morrey Type 2 may involve only a small fraction of the RN. This also becomes relevant for oblique fractures of the coronoid anterolateral facet. This potentially low affection of the proximal radioulnar joint may further support conservative treatment of these fractures if the radial head is intact or restored/replaced. While the morphometric data of this study have a low variability in terms of 95% confidence intervals, they also exhibit a large interindividual variability in terms of range. This large range is not caused by sex differences. Rather, our results showed a high correlation between measured and calculated parameters, suggesting that the large range may be caused by individual bone size. Because of the large range, even in seemingly simple fractures of the CP, cross-sectional imaging seems necessary to exclude an involvement of the proximal radioulnar joint (Fig. 4a, b). To not underestimate the involvement of the RN in anterolateral CP fractures in CT scans, cartilage must also be considered. However, cartilage thickness may be difficult or impossible to assess in CT scans. According to an anatomic study by Miyamura et al. [12], the thickness of the cartilaginous surface of the CP and RN is 2.20 mm ± 0.39 mm and 2.49 ± 0.55 mm, respectively. These values may be helpful when transferring our results to clinical practice.

### Strengths and limitations

The strengths of this study include the large sample size of specimens. Further, using high-resolution photographs instead of computed tomography scans or magnetic resonance imaging, we were able to accurately determine the actual cartilaginous surface of the RN. The mean age of the specimens was quite high with 79 years. Although specimens with visible signs of osteoarthritis were excluded during initial screening, our morphometric results may not be valid for younger patients.

## Conclusion

The radial notch of the ulna extends only to a small part to the coronoid process. Therefore, transverse or anterolateral fractures of less than 50% of the coronoid process may involve only a small portion of the proximal radioulnar joint. However, due to the wide variability, relying on lateral radiographs alone may not be sufficient to assess the involvement of the proximal radioulnar joint in this type of injury.

## Data Availability

Data are available from the corresponding author upon reasonable request.
